# Bulbar Muscle Weakness in the Setting of Therapeutic Botulinum Injections

**DOI:** 10.5811/cpcem.2018.8.39178

**Published:** 2018-09-28

**Authors:** Jacob Lentz, Daniel Weingrow

**Affiliations:** University of California, Los Angeles, Department of Emergency Medicine, Los Angeles, California

## Abstract

We present a case of new-onset bulbar muscle weakness in the setting of therapeutic botulinum injections for spasticity in a teenaged patient with cerebral palsy. Through a careful history, a systemic effect of the local injections was suspected, and the patient’s symptoms improved with a decrease in the dosing of the botulinum injections.

## INTRODUCTION

Though uncommon, systemic botulinum toxicity from local injections has been reported. With expanding use of therapeutic botulinum, it is important for emergency physicians (EP) to be aware of possible complications, the usual presentation, and management. As with foodborne botulism, recognition is key to preventing complications.

## CASE PRESENTATION

A 16-year-old girl with cerebral palsy, secondary to prenatal hypoxic brain injury, was brought into the emergency department (ED) by her parents because of progressive, nighttime gagging. The patient’s parents related that over the prior 10 months she had been having episodic and worsening episodes of choking on her own saliva at night while falling asleep and during sleep. These episodes would progressively worsen over five to six weeks and then improve. A few weeks later, the cycle would repeat.

During the day, the patient had no difficulty clearing secretions. The patient did not have fevers, chills, cough, or sputum production. She was taking her baseline diet. The patient’s speech was unchanged. The parents did endorse waxing and waning generalized weakness in the patient over the prior several months, the course of which tracked with the gagging. The patient’s only medication was famotidine, which she took by mouth. She had no allergies and had no other medical or surgical history. The patient was enrolled in high school, where she was doing well and had many friends. The parents strongly doubted any drug or alcohol use.

The patient’s parents related that her care was managed primarily by her neurologist with bi-monthly appointments. The choking episodes had, without fail, resolved by the time of each visit. There had been no changes in the patient’s health and no new illnesses or diagnoses for the preceding five years. The parents mentioned that at each visit for the prior two years the patient had been receiving local botulinum toxin injections in her arms and legs to relieve her muscle spasticity. The parents relayed that the neurologist had been increasing the dosing of botulinum over the prior year in an attempt to achieve better spasm control.

Physical exam revealed a friendly girl in no acute distress. No gagging or coughing was noted. Her vitals signs showed an oral temperature of 36.4 degrees Celsius, a heart rate of 71 beats per minute, a blood pressure of 115/78 millimeters of mercury, a respiratory rate of 14 breaths per minute, and an oxygen saturation of 100% on room air. The oropharynx was clear. Oral mucosa was moist without any obvious lesions. There was no drooling or difficulty clearing secretion, and she had an intact gag reflex. Extraocular movements were intact with no ptosis, and pupils were reactive to light. The patient’s tongue projected midline, and speech was at baseline per parents. There was no lymphadenopathy in the head or neck. The patient’s lungs were clear. The remainder of the exam was notable only for diffuse symmetrical muscle spasticity in the upper and lower extremities. A chest radiograph showed no consolidation or evidence of foreign body.

After careful clarification of the time course of the patient’s gagging episodes, it was suggested to the patient and her parents that she might have been having bulbar muscle weakness from botulinum toxin injections, which became noticeable when she was falling asleep, or asleep, and her muscle tone was already relaxed.

## DISCUSSION

The indications for and therapeutic uses of botulinum toxin continue to expand.^1^,^2^ Beyond its cosmetic effects, botulinum toxin has shown varying degrees of success in the treatment of symptoms in a variety of diseases, including Hirschsprung’s disease, cervical dystonia, spasticity, trigeminal neuralgia, amyotrophic lateral sclerosis, and hyperhidrosis.^3^,^4^ The near-ubiquity of therapeutic botulinum toxin injections belies the fact that it remains, based on dosage, the most lethal poison known.^5^ The estimated human median lethal dose (LD_50_) is 1.3–2.1 nanogram/kilogram (ng/kg) intravenously (IV) or intramuscularly (IM), 10–13 ng/kg inhaled, and 1 ng/kg oral.^6^,^7^ Given this, the probability that the EP will encounter adverse effects from its use continues to rise.

Botulinum toxin is produced by the gram-positive anaerobe *Clostridium botulinum*, a spore-forming bacterium that is ubiquitous in soil and water. The spores are usually dormant and quite hardy. The toxin, by contrast, is heat labile and is quickly destroyed by a few minutes of boiling water. The toxin is composed of light and heavy protein chains, and it exerts its effect in presynaptic nerve terminals by irreversibly preventing the releases of acetylcholine, resulting in flaccid muscle paralysis and anticholinergic autonomic effects.^6^

If a patient has been exposed to enough toxin, he will then develop bulbar palsies that can include dysphonia, dysarthria, dysphagia. Diplopia and ophthalmoplegia are also prominent findings, as well as dilated, non-reactive pupils. The patient may also endorse a sore throat and a dry mouth. Tendon reflexes and mental status are preserved, and the patient remains afebrile. Orthostatic hypotension or autonomic instability may be present. Flaccid extremity paralysis follows. Respiratory muscle weakness leading to respiratory failure is a potential life-threatening event in any patient with botulism.^6^–^8^

While the general practice is to give therapeutic botulinum toxin in the outpatient setting, adverse effects can occur. The therapeutic effect of botulinum injections occurs at the neuromuscular junction, which is the same mechanism by which the disease functions; therefore systemic toxicities suggestive of botulism are exaggerations of the therapeutic effect.^1^ One study of adverse events in therapeutic botulinum injections found that in such events, bulbar muscle weakness was present in 15% of patients, respiratory issues in 38%, ocular problems in 23%, bowel or bladder problems in 15%, and other muscle weakness in 15% of patients. Patient were also found to have infections from the injections 15% of the time (Table).^2^

Iatrogenic botulism is one of the seven types of botulism described by the World Health Organization (WHO).^8^ The other forms of botulism recognized by the WHO include the four classic distinct disease forms: foodborne botulism; infant botulism (for which nearly half of reported American cases have been in the state of California); wound botulism; and botulism of undetermined origin (in a patient over one year of age without a clear food or wound source). The WHO also recognizes two other potential forms of the disease, inhalational botulism (were it to be aerosolized as a weapon) and waterborne botulism (which is theoretical as water treatment inactivates the toxin).^6^,^8^

**CPC-EM Capsule**

What do we already know about this clinical entity?

Iatrogenic botulism is one of the seven types of botulism recognized by the World Health Organization and is caused by therapeutic botulinum toxin injections.

What makes this presentation of disease reportable?

The world’s deadliest toxin, botulinum toxin, is used for an expanding number of therapeutic purposes, and systemic effects, similar to other botulism entities, can be seen.

What is the major learning point?

Though uncommon, iatrogenic botulism should be kept on the differential for patients who present with muscle weakness and a history of botulinum toxin injections.

How might this improve emergency medicine practice?

After reviewing this case study and overview of botulism, emergency physicians may be more likely to consider therapeutic botulinum injections as the etiology of a patient’s muscle weakness.

Foodborne botulism often presents first with nondescript gastrointestinal symptoms, including nausea, vomiting, and abdominal pain and distension. These may occur at any point from a few hours to eight days after ingestion of the culprit item. Outbreaks of foodborne botulism have been caused by homemade foods, mishandled foods, and even industrially produced foods.^9^ Botulism has also been caused by consumption of prison-made wine (known as “pruno” or “hooch,” among other terms).^10^

Infant botulism is the most common form of botulism in the United States. It presents as constipation followed by flaccid neuromuscular paralysis. Cranial nerves are usually first affected, followed by peripheral and respiratory function. Honey, corn syrup, vacuum bag dust, and soil have been identified as sources of *C. botulinum* spores; it is believed that the infant gastrointestinal (GI) tract lack the bile acids and gut flora that normally inhibit *Clostridium* growth.^6^

Wound botulism presents identically to foodborne botulism, without GI symptoms. The culprit wounds are often in avascular areas or due to a crush injury, though most commonly they are due to intravenous drug use. A particularly high incidence has been noted among “skin popping” users of black tar heroin.^7^

Iatrogenic botulism, like wound botulism, can present with symptoms ranging from the very mild to impending respiratory compromise without GI symptoms.^6^ It is due to therapeutic injection of one of the three FDA-approved formulations of the toxin. A clinician should be suspicious of botulism based on a history that includes possible exposures via any of the previously described routes. Findings of cranial nerve palsies and extremity weakness support the diagnosis. Routine laboratory values and imaging, including brain computed tomography and magnetic resonance imaging, are nondiagnostic in botulism. Cerebral spinal fluid studies will also be nondiagnostic (though can have a slightly elevated protein count).^6^ Stool, serum and, as applicable, wound samples should be sent for anaerobic cultures and botulinum toxin detection. Consultation with a neurologist is also necessary to obtain electromyography, as botulism has a typical pattern of small units with subtle signs of denervation, and increased jitter with blocking.^1^ Patients with respiratory compromise may need to be intubated and mechanically ventilated.^9^

A clinician who suspects botulism should contact the state health department, and they will be referred to the Botulism Clinical Consultation Service at the Centers for Disease Control and Prevention (CDC).^9^ The CDC will send botulinum antitoxin from their stockpiles, and will also help coordinate epidemiologic research to quarantine possible outbreaks. Antitoxin does not reverse symptoms but rather prevents further progression. It is imperative that antitoxin be given as soon as possible if botulism is suspected, prior to confirmatory testing. Subsequent doses may be given at two-to-four hour intervals based on clinical disease progression.^6^,^9^

There is a human botulism immune globulin available for infant botulism, and it is available only from the California Department of Health Services Infant Botulism Treatment and Prevention Program. In wound botulism, surgical debridement is the primary therapy. Penicillin G should also be administered.^6^ Iatrogenic botulism can present along a wide spectrum of symptoms, and the management should be in relation to the severity of the symptoms.

## CONCLUSION

The patient was clinically stable and was monitored in the ED for several hours. The patient and her parents were offered admission, but they declined because they felt that the patient was on an “upswing” ahead of an appointment with her neurologist in two weeks. The patient was seen by her neurologist the next week, and he reduced the dose of botulinum toxin being used for her spasticity. The patient’s gagging did not return.

Documented patient informed consent and/or Institutional Review Board approval has been obtained and filed for publication of this case report.

## Figures and Tables

**Table:** Types of symptoms among patients with adverse effects from botulinum toxin injections for spasticity.^2^

Symptom category	Percentage
Muscle weakness	15
Oropharyngeal	15
Respiratory	38
Ocular	23
Bowel/bladder-related	15
Infection	15
